# A UWB-Ego-Motion Particle Filter for Indoor Pose Estimation of a Ground Robot Using a Moving Horizon Hypothesis

**DOI:** 10.3390/s24072164

**Published:** 2024-03-28

**Authors:** Yuri Durodié, Thomas Decoster, Ben Van Herbruggen, Jono Vanhie-Van Gerwen, Eli De Poorter, Adrian Munteanu, Bram Vanderborght

**Affiliations:** 1 Brubotics, Vrije Universiteit Brussel, 1050 Ixelles, Belgiumbram.vanderborght@vub.be (B.V.); 2 imec vzw, 3001 Leuven, Belgium; ben.vanherbruggen@ugent.be (B.V.H.); jono.vanhie@ugent.be (J.V.-V.G.); eli.depoorter@ugent.be (E.D.P.); adrian.munteanu@vub.be (A.M.); 3 IDLab, Universiteit Gent, 9000 Gent, Belgium; 4 ETRO Departement of Electronics and Informatics, Vrije Universiteit Brussel, 1050 Ixelles, Belgium

**Keywords:** sensor fusion, UWB, IMU, UWB pose estimation, robot pose control

## Abstract

Ultra-wideband (UWB) has gained increasing interest for providing real-time positioning to robots in GPS-denied environments. For a robot to act on this information, it also requires its heading. This is, however, not provided by UWB. To overcome this, either multiple tags are used to create a local reference frame connected to the robot or a single tag is combined with ego-motion estimation from odometry or Inertial Measurement Unit (IMU) measurements. Both odometry and the IMU suffer from drift, and it is common to use a magnetometer to correct the drift on the heading; however, magnetometers tend to become unreliable in typical GPS-denied environments. To overcome this, a lightweight particle filter was designed to run in real time. The particle filter corrects the ego-motion heading and location drift using the UWB measurements over a moving horizon time frame. The algorithm was evaluated offline using data sets collected from a ground robot that contains line-of-sight (LOS) and non-line-of-sight conditions. An RMSE of 13 cm and 0.12 (rad) was achieved with four anchors in the LOS condition. It is also shown that it can be used to provide the robot with real-time position and heading information for the robot to act on it in LOS conditions, and it is shown to be robust in both experimental conditions.

## 1. Introduction

Indoor positioning is an important step towards autonomous robots and can be approached in multiple ways [1]. One approach is to navigate without external infrastructure, as performed with a neural network-based approach in [2]. However, the task can be made easier by the knowledge of the position using external devices. Ultra-wideband (UWB) has gained significant interest over the past few years in the context of indoor positioning as can be seen from the increase in papers published on the topic [3], as well as the recent inclusion of UWB in the new iPhones. One of the major advantages of UWB is its use in GPS-denied environments while being lightweight, robust, and resilient against multipath fading and low in power consumption [4]. Much research is focused solely on accurately localizing a tag [5], e.g., in [6], where machine learning was used on Channel Impulse Response.

Recently, UWB has gained attention in the context of localizing and controlling a robotic platform in real time. However, to use UWB to control a robotic platform, the pose (the combination of location and orientation) has to be available in real time. UWB itself has a dm-level localization error and does not provide orientation, which is generally mitigated by using it with other modalities or by using multiple tags.

A first strategy exists for trying to estimate the location [7,8,9,10] and, sometimes, the orientation [11,12,13,14,15] using UWB in combination with a (low-cost) IMU [7,8,9,11,12,13,14] or odometry [10,15]. A second strategy exists for solving a Simultaneous Localization and Mapping (SLAM) problem combining UWB with visual inputs (e.g., camera [16,17,18] or LiDAR [19,20]), sometimes in combination with an IMU [17,18] or odometry.

This paper focuses on the first group of algorithms, where the pose (position and orientation) is estimated using UWB in fusion with ego-motion estimators. Ego-motion estimators are any kind of sensor that is part of the robot and can track the motion of the robot, e.g., an IMU, wheel encoders, etc. In Table 1, these algorithms are compared. Multiple challenges arise in this case.

The first challenge comes from the intrinsic lack of orientation information coming from UWB measurements. This challenge was overcome in [12,15] by using multiple tags that create a local reference frame connected to the platform while operating in a multi-anchor environment. Ref. [14] made use of the shape of the UWB antenna to estimate the orientation of the tag in a multi-anchor setup. This orientation estimation was used to correct the orientation information received from a gyroscope. A drawback of this approach is that knowledge about the radiation pattern of the antenna is required. The authors overcame this by training a Gaussian process model. All other algorithms using a single UWB tag combine a gyroscope with magnetometer measurements to obtain the orientation [7,8,9,10,11,13]. Measuring the magnetic field in complex indoor environments is challenging due to magnetic distortion [14] and, thus, is not an ideal solution.

Another challenge arises from the coarse error in the UWB measurements. Refs. [9,10] overcame this by using a moving horizon estimator, which averages the measurements during a certain time to decrease the uncertainty. It was used with a small local time horizon to mimic the actual value as a filter. Ref. [7] also used an averaging window to obtain the real velocity while working with a constant velocity assumption. Most research, however, did not particularly try to mitigate the coarse error from the UWB measurements [8,11,13,15]. The error of the UWB is, in most cases, considered as Gaussian, but as pointed out by [10,21], the error model is more likely to be heavy-tailed skewed due to multipath and Non-Line-Of-Sight (NLOS) conditions. Some algorithms made use of outlier rejection to increase the UWB accuracy [10].

As pointed out by [22], ego-motion measurements suffer from drift due to the integration of the measurements during the dead-reckoning phase. Moreover, IMUs suffer from a slowly changing bias that affects the accelerometer and gyroscope in the long run [21]. It is common to neglect these small changing biases provided that, with a calibrated IMU and a limited operation time, the bias stays limited. However, Ref. [13] explicitly estimated this bias in the proposed algorithm. Ref. [9] did not explicitly calculate the bias, but added a term to the cost function in his algorithm, which was proportional to the velocity, thereby keeping the drift in check. Ref. [11] explicitly modeled the slowly changing bias using a Wiener process for the accelerometers.

The last challenge lies in fusing the ego-motion measurements with UWB. The goal is to arrive at an algorithm that works at run-time while reducing the error on the pose as much as possible. Most kept the location and orientation as separate issues where a Kalman filter (KF) was used to fuse the gyroscope with the magnetometer [7,8,9,10,11,13]. A separate algorithm was used to fuse the odometry data with the UWB measurements to estimate the position. In most cases, an extended Kalman filter (EKF) was used to fuse the odometry with UWB [7,13]. Ref. [15] used Multiplicative EKF. Ref. [8] used a Long Short-Term Memory neural network to fuse the data and compared it to EKF and Unscented Kalman filter. Although they achieved better results, the computational load was considerably higher. Another strategy is to use a gradient descent algorithm on a cost function, as was performed in [9,10], achieving good results in a few iterations. Ref. [11] proposed a particle filter (PF) to estimate the bias on the accelerometer and the position. Herein, the IMU served as a model to predict the position of the platform, which was corrected by the UWB measurements, yet the PF required 100.000 particles to operate and had a considerable influence on the computational load. Ref. [14], on the other hand, only estimated the orientation and not the position using an invariant EKF, where the gyroscope was corrected by their Gaussian process model for orientation.

The novelty presented in this paper is a UWB-ego-motion particle filter for indoor pose estimation of a ground robot using a moving horizon hypothesis, which includes the following contributions:

- Estimating the heading and position of a ground robot fusing ego-motion with UWB for a single-tag multi-anchor setup.
- Reductions to separate the influence of the bias from the model equations to reduce the number of operations and reduce the computational load.
- The algorithm runs in real time and has been used to control a robot in the lab.

A video of the algorithm and it is performance can be found at https://youtu.be/3DBzxUPCwmE.

In Section 2.2, the particle filter algorithm is derived. In Section 3, the experiments will be discussed. Using the algorithm to control the trajectory of the robot in real life is detailed in Section 3.3. Finally, the conclusion is drawn in Section 5. In the following section, the nomenclature and setup will be addressed first.

## 2. Materials and Methods

### 2.1. Nomenclature

In Table 2, the variables that will be used in this paper are listed.

**Figure 1:**
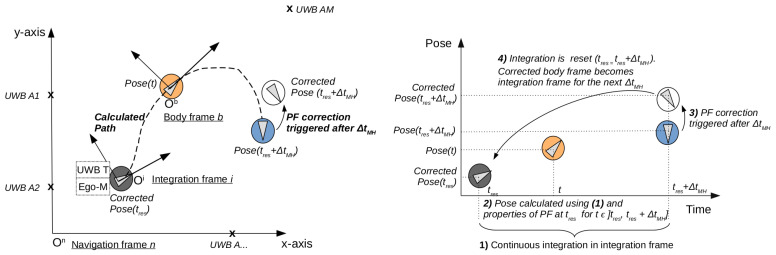
Geometrical axis (**top** figure) and time axis (**bottom** figure) with reference frames and events in the algorithm. The pose at the start of the moving horizon period (dark grey), the current calculated pose (orange), the calculated end pose at the end of the moving horizon period (blue), and the corrected pose (white) are shown. After one moving horizon period, the corrected pose (white) becomes the new start pose (dark grey).

A superscript *r* will indicate in which reference frame a variable is expressed. Three reference frames are used, as can be seen in Figure 1. The navigation frame *n* is the frame in which the movement of the robot needs to be expressed and is fixed to the environment. The body frame *b* is attached to the robot. The integration frame *i* is fixed during a moving horizon period Δ*t_MH_*; it is the body frame at the moment the moving horizon period starts and is updated every moving horizon period.

A subscript *m* will indicate the modality by which a variable has been measured or derived. The different modalities are acc, gyr, odom and uwb for, respectively, accelerometer, gyroscope, odometry, and UWB. The *pf* subscript is given to the variables of the weighted particle from the particle filter (PF) at the reset time *t_res_*.

### 2.2. Algorithm Overview

A PF will be used to correct the integrated pose from the ego-motion estimation with the measurements from UWB. In Figure 1, the theoretical setup is shown. An environment is equipped with A1, A2, …, AM UWB anchors, and the location of the anchors is known in the navigation frame *n*. A robot equipped with an ego-motion modality (wheel encoders, IMU, …) and an UWB tag moves around in a horizontal plane. The start pose (position and orientation) of the robot is shown with the dark grey circle and marks the reference frame with origin $O^{i}$. The start pose is given by the pose of the robot at the start of a moving horizon period at time $t_{res}$. The current calculated robot pose is shown with the orange circle and marks the local reference frame attached to the body of the robot with origin $O^{b}$. This pose is calculated from the integration of the ego-motion sensors in the integration frame *i*. Using the weighted particle, calculated at the start of this moving horizon period, the ego-motion integration is transformed from the integration frame *i* to the navigation frame n. The robot continues its movement until the moving horizon period $\Delta t_{MH}$ has passed. The calculated pose at the end of this time frame is shown in blue. During this, time drift occurred. The particle filter will correct the pose using the UWB measurements obtained during the same moving horizon time period. The corrected pose is shown in white and will mark the new integration frame *i* from which a new integration period can start.

**Figure 2:**
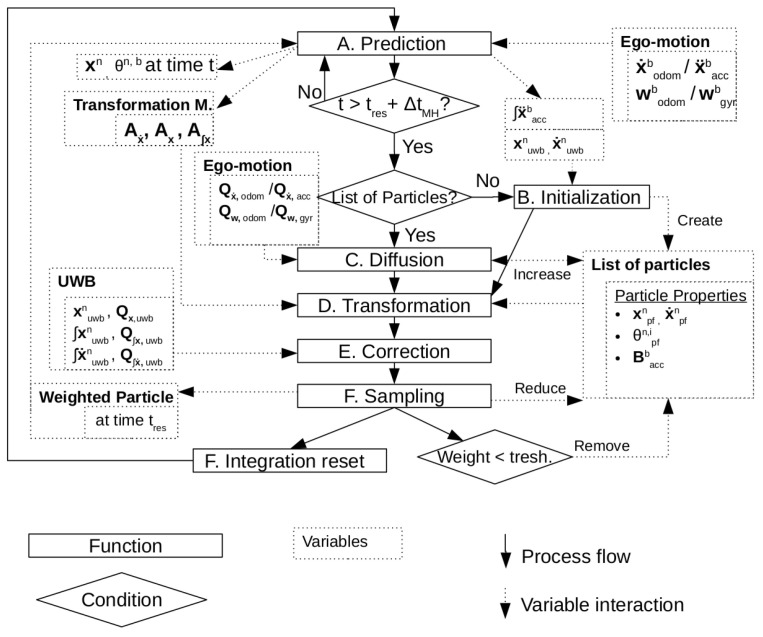
Scheme of the proposed algorithm. The boxes represent functions that are discussed in the sections below. The dotted boxes represent the variables involved in each function, while the diamonds represent important decisions.

The complete scheme describing the algorithm is shown in Figure 2. A more detailed explanation per step is given in the next subsections.

### 2.3. The Particle Filter

The states $x\left(t\right)\in R^{10}$ are given by:

(1) $$x\left(t\right)={\left[\begin{array}{cccc} x_{acc}^{n}\left(t\right) & {\dot{x}}_{acc}^{n}\left(t\right) & \theta^{n,b}\left(t\right) & B_{acc}^{b}\left(t\right) \end{array}\right]}^{t},$$

where $x_{acc}^{n}\left(t\right)$, ${\dot{x}}_{acc}^{n}\left(t\right)$, and $\theta^{n,b}\left(t\right)$ are the position, velocity, and orientation of the robot at the start of the integration frame *i*, respectively. $B_{acc}^{b}\left(t\right)$ is the bias on the acceleration of the accelerometer. The filter is initialized, as detailed in Section 2.3.2 (B), after the first integration. The input $u\left(t\right)\in R^{4}$ is given by:

(2) $$u={\left[\begin{array}{cc} {\ddot{x}}_{acc}^{n}\left(t\right) & \omega_{gyr}^{b}\left(t\right) \end{array}\right]}^{t},$$

where ${\ddot{x}}_{acc}^{n}\left(t\right)$ is the measured acceleration vector and $\omega_{gyr}^{b}\left(t\right)$ is the measured rotation around the *z*-axis of the robot by the IMU. This input is integrated as explained in Section 2.3.1 (A) during the integration window. In this step, the transformation matrices $A_{\ddot{x}}$, $A_{\dot{x}}$, and $A_{x}$ are calculated as explained in that section. The input covariance $Q\left(t\right)\in R^{4\times 4}$ is given by:

(3) $$Q\left(t\right)=\mathrm{diagonal}\left(\sigma_{acc}^{2},\sigma_{acc}^{2},\sigma_{acc}^{2},\sigma_{w^{b},gyr}^{2}\right),$$

where $\sigma_{acc}^{2}$ and $\sigma_{w^{b},gyr}$ are the measurement noise on the acceleration and rotational speed of the IMU. These are used in the diffusion step as explained in Section 2.3.3 (C) to represent the distribution of the particles. The measurement, $z\left(t\right)\in R^{3}$, is given by:

(4) $$z\left(t\right)=x_{uwb}^{n}\left(t\right),$$

where $x_{uwb}\left(t\right)$ is the UWB-measured position. The covariance $R\in R^{3\times 3}$ is given by:

(5) $$R\left(t\right)=\mathrm{diagonal}\left(\sigma_{x^{n},uwb}^{2},\sigma_{x^{n},uwb}^{2},\sigma_{x^{n},uwb}^{2}\right),$$

where $\sigma_{x^{n},uwb}$ is the standard deviation on the measured position in one axis. The measurements do not directly trigger a correction; this is only performed after the integration window is finished. In this case, the the transformation matrices as calculated in Section 2.3.1 (A) are applied to all particles as explained in Section 2.3.4 (D), after which all measurements within the integration time are used, as explained in Section 2.3.5 (E). Finally, sampling is applied as detailed in Section 2.3.6 (F).

#### 2.3.1. (A) Prediction

The prediction will be given by the integration of the ego-motion sensor. The pose of the new integration frame *i* in the navigation frame *n* is given by the position of the weighted particle $x_{pf}^{n}$. We start from the equations derived in [21] for the accelerometer and gyroscope:

(6) $$\begin{array}{cc} {\ddot{x}}_{acc}^{n}+g^{n} & =R^{b,n}\left({\ddot{x}}_{acc}^{b}-B_{acc}^{b}\right)+e_{acc}^{b} \end{array}$$

(7) $$\begin{array}{cc} w_{gyr}^{n} & =w_{gyr}^{b}-B_{gyr}^{b}+e_{gyr}^{b}, \end{array}$$

where $g^{n}$ is the gravitation vector as expressed in the navigation frame *n*. Since the robot is moving in a horizontal plane, the gravitation vector will be ignored. Leaving out the noise term and using the rotation matrix operations, Equation (6) becomes:

(8) $$\begin{array}{cc} {\ddot{x}}_{acc}^{n} & =R^{n,i}R^{i,b}{\ddot{x}}_{acc}^{b}-R^{n,i}R^{i,b}B_{acc}^{b}. \end{array}$$

Rearranging the terms of Equation (8) in a matrix yields:

(9) $$\begin{array}{cc} {\ddot{x}}_{acc}^{n} & =R^{n,i}\left[\begin{array}{cc} R^{i,b}{\ddot{x}}_{acc}^{b} & R^{i,b} \end{array}\right]\left[\begin{array}{c} 1\\ -B_{acc}^{b} \end{array}\right]\\ \; & =R^{n,i}A_{\ddot{x}}{\hat{B}}_{acc}^{b}, \end{array}$$

where $R^{n,i}$ and ${\hat{B}}_{acc}^{b}={\left[\begin{array}{cc} 1 & -B_{acc}^{b} \end{array}\right]}^{\tau }$ are considered constant during a moving horizon time period $\Delta t_{MH}$. Allow integration for $t\in \;]t_{res},t_{res}+\Delta t_{MH}]$:

(10) $$\begin{array}{cc} {\dot{x}}_{acc}^{n}= & {\dot{x}}_{pf}^{n}+R^{n,i}A_{\dot{x}}{\hat{B}}_{acc}^{b} \end{array}$$

(11) $$\begin{array}{cc} x_{acc}^{n}= & x_{pf}^{n}+\left(t-t_{res}\right){\dot{x}}_{pf}^{n}+R^{n,i}A_{x}{\hat{B}}_{acc}^{b}\\ \; & \;\mathrm{with}\;A_{\dot{x}}=\int_{t_{res}}^{t}A_{\ddot{x}}dt\;\mathrm{and}\;A_{x}=\int_{t_{res}}^{t}A_{\dot{x}}dt, \end{array}$$

where $x_{pf}$ and ${\dot{x}}_{pf}$ are the position and speed of the weighted particle and represent the starting conditions of the integration. Taking the moving average for Equations (10) and (11) between $t\in \;]t_{res},t_{res}+\Delta t_{MH}]$ gives:

(12) $$\begin{array}{cc} {\bar{\dot{x}}}_{acc}^{n}= & {\dot{x}}_{pf}^{n}+R^{n,i}{\bar{A}}_{\dot{x}}{\hat{B}}_{acc}^{b} \end{array}$$

(13) $$\begin{array}{cc} {\bar{x}}_{acc}^{n}= & x_{pf}^{n}+\frac{\Delta t_{MH}}{2}{\dot{x}}_{pf}^{n}+R^{n,i}{\bar{A}}_{x}{\hat{B}}_{acc}^{b}. \end{array}$$

Getting rid of the gyroscope bias is more difficult. Hence, it will be assumed that the gyroscope is calibrated and the effect of the slowly changing bias stays within the error of the gyroscope. Generally, $R^{i,b}$ at time $t_{n}$ can be calculated using the discretized measured angular velocity with:

(14) $$\begin{array}{cc} R^{i,b}\left(t_{n}\right) & =R^{i,b}\left(t_{res}\right)\prod_{j=0}^{j=n}e^{dtw_{gyr}^{b}\left(t_{j}\right)}. \end{array}$$

Since the integration is reset after each moving horizon period, $R^{i,b}\left(t_{res}\right)=I$. In the case of a robot moving in a horizontal plane, Equation (14) becomes:

$$\begin{array}{cc} w_{gyr}^{b}= & {\left[\begin{array}{ccc} 0 & 0 & w_{gyr}^{b} \end{array}\right]}^{t} \end{array}$$

(15) $$\begin{array}{cc} \theta^{i,b}\left(t\right)= & \int_{t_{res}}^{t}w_{gyr}^{b}\left(t\right)dt. \end{array}$$

$R^{i,b}$ can be calculated form $\theta^{i,b}\left(t\right)$ and $\theta^{n,b}=\theta_{pf}^{n,i}+\theta^{i,b}$, where $\theta_{pf}^{n,i}$ will be kept by the particles and from which $R^{n,i}$ can be calculated in Equation (9).

Similar deduction can be made when not ${\ddot{x}}_{acc}^{b}$, but ${\dot{x}}_{odom}^{b}$ is provided. During the integration step only, $A_{\dot{x}}$, $A_{x}$, and $A_{\int x}$ will be calculated gradually until a period $\Delta t_{HM}$ has passed. The pose of the robot will be estimated continuously using Equations (11) and (15), where the weighted particle is used. The weighted particle represents the weighted sum of all particles in the PF where the weight is given by the particle likelihood and as such represents the best estimate of the filter. As can be seen in Equation (11), $x_{pf}$ and ${\dot{x}}_{pf}$ represent the most likely position and speed at the starting conditions of the integration interval. The last term of the equation represents the contribution due to acceleration in the position estimation within the integration interval.

#### 2.3.2. (B) Initialization

Once the integration over a period $\Delta t_{MH}$ has been completed, 2 options exist: either a list of particles exists and the diffusion step can start or no list is available and a list has to be initialized. In the latter case, an initial estimate has to be made for the properties of the particles $x_{pf}^{n}$, ${\dot{x}}_{pf}^{n}$, $\theta_{pf}^{n,e}$, and $B_{acc}^{b}$.

For $x_{pf}^{n}$ and ${\dot{x}}_{pf}^{n}$, UWB will be used with an uncertainty bound equal to the uncertainty on these values.

For the orientation, $\theta_{pf}^{n,e}$ a uniform distribution will be taken from the interval ]−π,π].

At last, for the bias on the accelerometer, a uniform distribution will be taken from the interval $\left[{\bar{\ddot{x}}}_{acc}^{b}-a_{max},{\bar{\ddot{x}}}_{acc}^{b}+a_{max}\right]$. ${\bar{\ddot{x}}}_{acc}^{b}$ is the moving average of the measured acceleration in the body frame *b*, and $a_{max}^{\tau }=a_{max}\left[\begin{array}{ccc} 1 & 1 & 1 \end{array}\right]$ and $a_{max}$ are the theoretical maximum acceleration of the robot.

#### 2.3.3. (C) Diffusion

During the integration step, the uncertainties were omitted to reduce the number of operations. However, these uncertainties have to be taken into account in the particle filter. To avoid difficult integration, the strategy would be to generate particles around the calculated particles that represent the uncertainty after an integration period of $\Delta t_{MH}$. For this purpose, each particle will obtain n children for which the values of $x_{pf}^{n}$, ${\dot{x}}_{pf}^{n}$, and $\theta_{pf}^{n,e}$ will be drawn from a distribution around the original value from the parent particle. For $x_{pf}^{n}$, starting from Equations (14) and (15) and knowing the gyroscope has been calibrated, the variance on the orientation becomes:

(16) $$\begin{array}{c} \sigma_{\theta^{e,b},gyr}^{2}=\left(\frac{\Delta t_{MH}}{f_{s}}\right)\sigma_{w^{b},gyr}^{2}, \end{array}$$

where $\sigma_{w^{b},gyr}^{2}$ is given by the gyroscope and $f_{s}$ is the sampling frequency of the gyroscope. In order to find the uncertainty bounds of the acceleration in the navigation frame *n* from the acceleration in the body frame *b*, it will be assumed that the error on the accelerometer is unrelated to the error on the gyroscope. In this case, the variance of the acceleration of the robot moving in a horizontal plane becomes:

(17) $$\begin{array}{c} \sigma_{{\ddot{x}}_{acc}^{n}}^{2}={\left({\ddot{y}}_{acc}^{n}\right)}^{2}\sigma_{\theta^{e,b},gyr}^{2}+\sigma_{acc}^{2} \end{array}$$

(18) $$\begin{array}{c} \sigma_{{\ddot{y}}_{acc}^{n}}^{2}={\left({\ddot{x}}_{acc}^{n}\right)}^{2}\sigma_{\theta^{e,b},gyr}^{2}+\sigma_{acc}^{2}, \end{array}$$

where $\sigma_{acc}^{2}$ is the variance in the acceleration given by the accelerometer. Since ${\ddot{y}}_{acc}^{n}$ and ${\ddot{x}}_{acc}^{n}$ are a function of time and need to be calculated, they will be replaced with the theoretical maximum acceleration of the robot $a_{max}$. This will overestimate the error, which is not an issue as long as convergence is reached. Finally, one can derive the following uncertainties for the velocity and position:

(19) $$\begin{array}{c} \sigma_{{\dot{x}}_{acc}^{n}}^{2}={\left(a_{max}\right)}^{2}{\left(\frac{\Delta t_{MH}}{f_{s}}\right)}^{2}\sigma_{w^{b},gyr}^{2}+\left(\frac{\Delta t_{MH}}{f_{s}}\right)\sigma_{acc}^{2} \end{array}$$

(20) $$\begin{array}{c} \sigma_{x_{acc}^{n}}^{2}={\left(a_{max}\right)}^{2}{\left(\frac{\Delta t_{MH}}{f_{s}}\right)}^{3}\sigma_{w^{b},gyr}^{2}+{\left(\frac{\Delta t_{MH}}{f_{s}}\right)}^{2}\sigma_{acc}^{2}. \end{array}$$

Lastly, the slowly changing bias $B_{acc}^{b}$ still has to be estimated. For this, a Wiener process with slow rate T will be used as proposed by [11].

#### 2.3.4. (D) Transformation

In this step, all previously generated particles will undergo the transformations as described in Equations (10)–(13). The values of the particle filter are updated as well in this step:

(21) $$\begin{array}{cc} {\dot{x}}_{pf}^{n}= & {\dot{x_{pf}^{n}}}^{*}+R^{n,e}A_{\dot{x}}{\hat{B}}_{acc}^{b} \end{array}$$

(22) $$\begin{array}{cc} x_{pf}^{n}= & {x_{pf}^{n}}^{*}+\left(\Delta t_{MH}\right){\dot{x_{pf}^{n}}}^{*}+R^{n,e}A_{x}{\hat{B}}_{acc}^{b}. \end{array}$$

The * indicates that it is the previous particle value.

#### 2.3.5. (E) Correction

In the correction step, UWB will be used to correct Equations (11)–(13) for each particle. First, the 3D position $x_{uwb}^{n}$ is calculated from the UWB ranges using the linear least squares method as described in [23]. From that, the moving average position ${\bar{x}}_{uwb}^{n}$ and the moving average velocity ${\bar{\dot{x}}}_{uwb}^{n}$ are calculated during the moving horizon duration $\Delta t_{MH}$:

(23) $$\begin{array}{cc} {\bar{x}}_{uwb}^{n}= & \frac{1}{\Delta t_{MH}}\int_{t-\Delta t_{MH}}^{t}x_{uwb}^{n}dt \end{array}$$

(24) $$\begin{array}{cc} {\bar{\dot{x}}}_{uwb}^{n}= & \frac{1}{\Delta t_{MH}}\left(x_{uwb}^{n}\left(t\right)-x_{uwb}^{n}\left(t-\Delta t_{MH}\right)\right). \end{array}$$

It will be assumed that the error on $x_{uwb}^{n}$ is zero mean Gaussian with a variance $\sigma_{x^{n},uwb}^{2}$, given by the setup. From this, the variance on ${\bar{x}}_{uwb}^{n}$ and ${\bar{\dot{x}}}_{uwb}^{n}$ can be calculated:

(25) $$\begin{array}{cc} \sigma_{{\bar{x}}^{n},uwb}^{2} & =\frac{1}{\Delta t_{MH}f_{uwb}}\sigma_{x^{n},uwb}^{2} \end{array}$$

(26) $$\begin{array}{cc} \sigma_{{\bar{\dot{x}}}^{n},uwb}^{2} & =\frac{2}{{\left(\Delta t_{MH}\right)}^{2}}\sigma_{x^{n},uwb}^{2}, \end{array}$$

with $f_{uwb}$ the update rate of the UWB setup.

Using ${\bar{x}}_{uwb}^{n}$ and ${\bar{\dot{x}}}_{uwb}^{n}$ on top of $x_{uwb}^{n}$ will greatly restrict the number of likely particles and, thus, allow using fewer particles.

#### 2.3.6. (F) Sampling and Integration Reset

Since more particles have been generated during diffusion, in this step, the number of particles will be reduced again. To limit the number of calculations and keep every possibility as long as possible, it was chosen to use multinomial sampling to bring back the particle population to the initial number of samples. During this process, a weighted particle will be calculated. The properties $x_{pf}^{n}$, ${\dot{x}}_{pf}^{n}$, $\theta_{pf}^{n,e}$, and $B_{acc}^{b}$ of this weighted particle will be used in the integration step.

After the resampling has taken place, all transformation matrices $A_{\dot{x}}$, $A_{x}$, and $A_{\int x}$ are reset and the integration can start a new moving horizon period $\Delta t_{MH}$.

### 2.4. Additional Points

#### 2.4.1. Particle Depletion

By leaving the propagation of the error until after the integration and limiting the number of particles or due to physical effects that are not captured by the uncertainties like slip, shock, or NLOS, particle depletion can kick in. For this reason, the weight of the filtered particles is monitored, and when particle depletion is detected, the current particle list is removed and a new one is initialized.

#### 2.4.2. Operation Reduction

Traditionally a PF would have to apply the prediction integration to each particle. This would mean an enormous amount of operations proportional to the number of particles. Using the proposed method, the number of operations is drastically reduced. It can be estimated that, when the sampling frequency of the ego-motion modality is 50 Hz, $\Delta t_{MH}$ is 1 s and the number of particles is 1000, while the number of operations is reduced from 900 k to 80 k operations. On top of this, the algorithm used in the experiments only uses 100 to 1000 particles to track the robot, as opposed to the 100.000 from [11].

### 2.5. Limitations

#### 2.5.1. Moving Horizon Duration Trade-Off

One can see from Equations (25) and (26) that the higher the moving horizon duration $\Delta t_{MH}$ is, the more accurate UWB becomes; however, from Equations (16), (19) and (20), one can understand that the higher $\Delta t_{MH}$ becomes, the more particles are needed to represent the uncertainty. For each $\Delta t_{MH}$, the transformations are calculated for all particles, resulting in much computational effort, so it is best not to take it too small.

#### 2.5.2. Gyroscope Calibration

When the bias on the gyroscope increases too much, the gyroscope will need to be calibrated. This requires the robot to stand still for a certain amount of time. This is only a small inconvenience compared to the full calibration of an IMU. It might be possible to generalize the idea and separate the bias of the gyroscope, as has been performed for the accelerometer, but this is not part of this paper.

#### 2.5.3. Unobservability of Orientation

When standing still for a long time, the uncertainty of the orientation will grow since it cannot be directly measured from UWB.

#### 2.5.4. UWB Position vs. Ranges

For now, the algorithm only works with UWB positions and not ranges. This has the drawback of only Gaussian error models being able to be used for UWB. It might be possible to update the algorithm to work with ranges, but this is not part of this paper.

## 3. Results

The algorithm was first tested offline and later used in a real-time experiment to control the pose of a moving robot.

### 3.1. Experimental Setup

The experiments were conducted in a large room equipped with Motion Capture (mo-cap) technology from Qualysis, Göteborg, Sweded and UWB. The UWB system consists of fixed Wi-Pos [24] anchor nodes in a cubic constellation. This Wi-Pos platform expands the possibilities of the DW1000 transceiver from Qorvo, Greensboro, NC, USA, with a wireless long-range backbone. The distance between the tag and anchor is determined with double-sided two-way ranging to eliminate the influence of clock drift. The location of the anchors used is given in Table 3.

**Figure 3:**
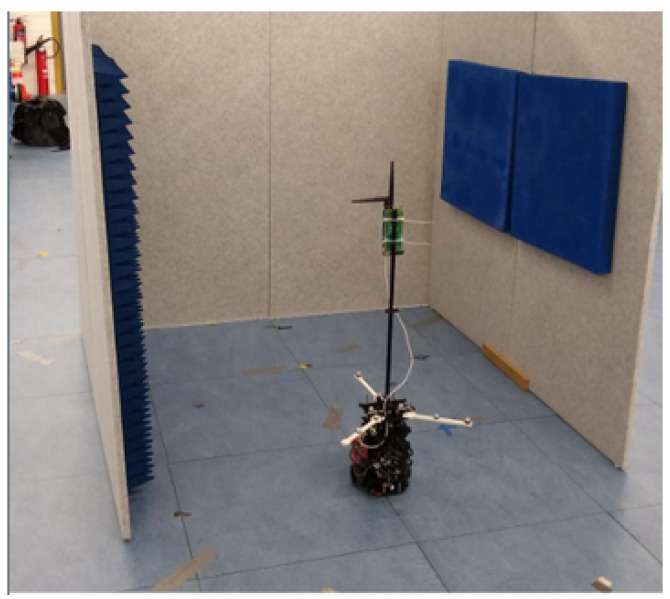
A Turtlebot3 equipped with mo-cap markers, an UWB tag, and ego-motion modalities is used to validate the particle filter to estimate the pose of the robot in different operating conditions.

**Figure 4:**
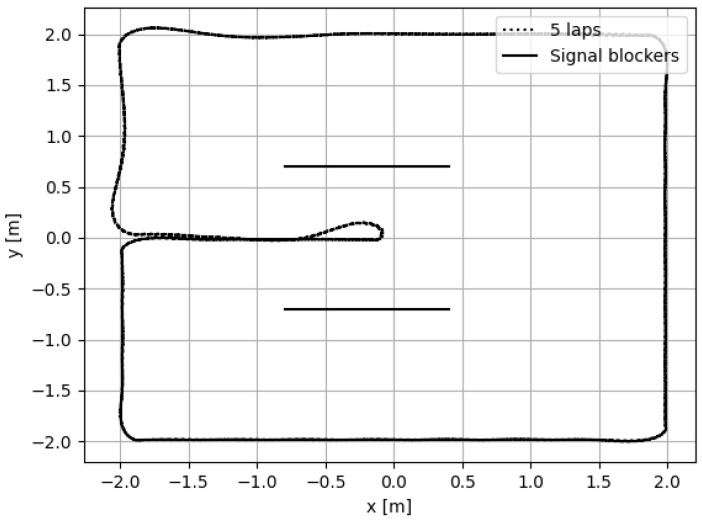
The experimental setup: with the trajectory and the non-line-of-sight object. The robot follows five U-shaped trajectories controlled by the mo-cap system.

A Turtlebot3 from ROBOTIS Co., Ltd., Seoul, Republic of Korea, was upgraded to include mo-cap markers and a UWB tag, as can be seen in Figure 3, and software was written to control the trajectory of the robot using mo-cap as feedback. Mo-cap will also provide mm-level accuracy on the ground truth. Table 4 shows the models of the sensors used for the evaluation of the proposed method. One continuous trajectory in the shape of a U was defined. The trajectory consists of sharp corners, which induce a particularly hard condition on the ego-motion sensors. The trajectory can be seen in Figure 4. Each experiment consisted of five laps. It took about 500 s to complete all five laps. Three experiments were conducted:

1. Modalities: In the first experiment, the different modalities were tested, and Table 5 lists the modalities used. The experiment was conducted in the LOS with the first four anchors. The next two experiments were performed with the better performing modality.
2. Influence of NLOS: In the second experiment, the influence of NLOS was tested. For this, the first experiment was repeated, but two walls were placed, which occluded the LOS for different anchors on the trajectory.
3. Influence of the number of anchors: In the last experiment, the influence of the number of anchors was tested, by redoing the first experiment, but with seven anchors instead of four.

For all three experiments, a cumulative distribution function (CDF) was used for both the error on the position and the orientation. The *y*-axis of a CDF shows the probability of the error to be less than or equal to the value on the *x*-axis.

### 3.2. Offline Tracking

The algorithm was first validated offline and compared to the reference modalities. Table 6 shows the values of the different algorithmic parameters that were used during these experiments.

**Figure 5:**
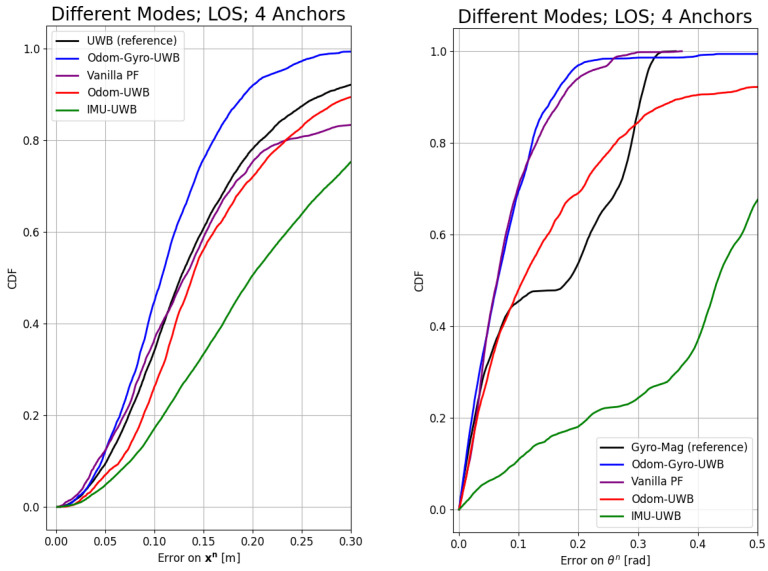
Cumulative distribution function of the error on the position (**left**) and orientation (**right**) for 4 anchors in the line-of-sight condition. In red is the Odom–UWB modality, in blue the Odom–Gyro–UWB modality, and in green the IMU–UWB modality. In black, the direct position from the UWB is shown, as well as the orientation from the Gyro–Mag modality. In purple, the vanilla PF is shown. The Odom–Gyro–UWB modality performs the best for the position and will be used in the next experiments to investigate the influence of the NLOS and the number of anchors. The vanilla PF and the Odom–Gyro–UWB modalities perform almost equally on the estimation of the orientation. To understand the effects of the proposed method in the NLOS, the vanilla PF will be used in the NLOS experiment as well. The IMU–UWB modality performs the worst, yet the error on the orientation stays within $\frac{\pi }{2}$.

In Figure 5, the CDF is shown of the error on the position and orientation with respect to the ground truth as measured by mo-cap for the different modalities using four anchors in the LOS. In this experiment, the Odom–Gyro–UWB modality (blue) performed the best and outperformed all other modalities. Therefore, this modality was used for the analysis of the NLOS and the number of anchors. Moreover, the sensors of this modality were used for the vanilla particle filter implementation shown in purple.

**Figure 6:**
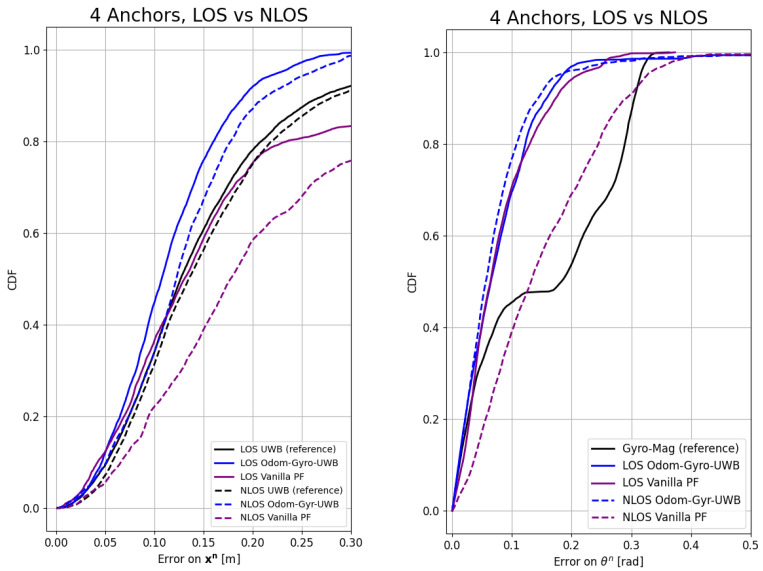
Cumulative distribution function of the error on the position (**left**) and orientation (**right**) for the Odom–Gyro–UWB modality in the Line of Sight (LOS) and the non-LOS (NLOS). In the NLOS, the reference, UWB (in black), seems only slightly affected. The position estimation seems slightly affected by the NLOS, but the orientation seems unaffected for the proposed method. The vanilla PF (in purple) deteriorates heavily under the NLOS conditions for both the orientation and the position, highlighting the strength of the proposed method.

**Figure 7:**
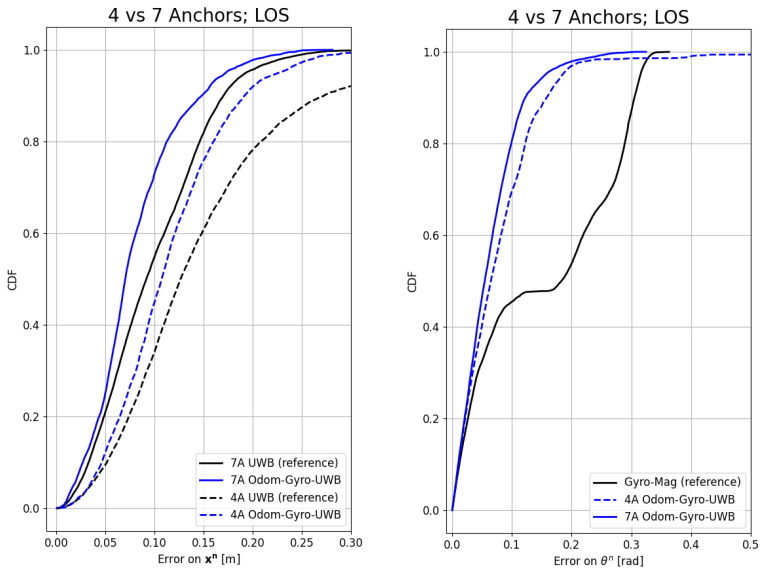
Cumulative distribution function of the error on the position (**left**) and orientation (**right**) for the Odom–Gyro–UWB modality with a different number of anchors. The performance increases the most when fewer anchors are used, but notable improvement can still be seen when using more anchors.

Figure 6 and Figure 7 show the influence of the NLOS and the number of anchors on the CDF, respectively. Table 7 and Table 8 summarize the results of the experiments for the P50 (the chance of the error being smaller than this value is 50%), P90, RMSE, and maximum error values of the different algorithms and environments under test. In the tables, some results are highlighted. The algorithm took around 75 s to calculate almost 500 s of data in the offline experiment.

### 3.3. Real-Time Control Experiment

**Figure 8:**
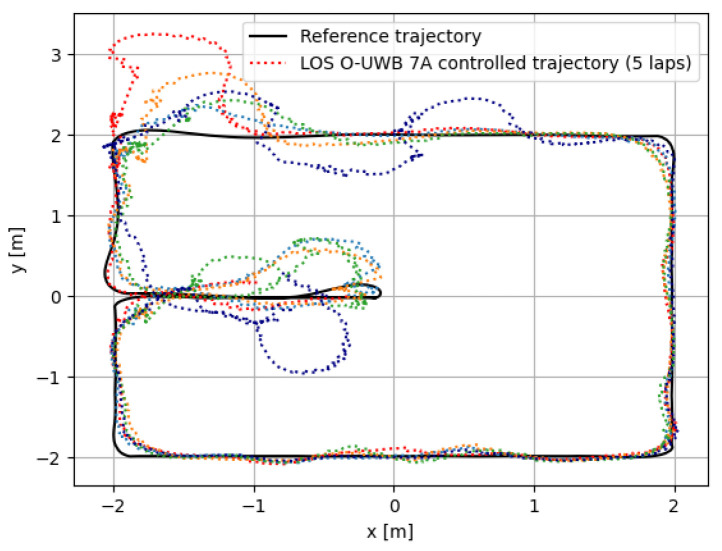
Robot controlled in real time with the UWB pose estimation algorithm. The expected trajectory that the robot performed in the offline experiment with the mo-cap as feedback is shown in black. The dotted color lines show each lap when the algorithm is used as feedback to the trajectory control instead of the mo-cap. Each color represents one of the 5 laps performed with the algorithm used as pose control.

The algorithm was also tested as the input to the pose control of the robot in real time. In this experiment, the robot was set to perform the same trajectory as with the offline experiment. The resulting trajectory is shown in Figure 8.

## 4. Discussion

### 4.1. Offline Tracking Performance

#### 4.1.1. Modalities

The algorithm performs robustly with different types of ego-motion modalities in estimating the robot’s pose, as can be seen in Figure 5. The IMU–UWB modality (green) performed worse than the other two modalities. The main reason is probably the strong noise on the inexpensive accelerometers of the Turtlebot3. The values of $\pm 2.5\frac{m}{s^{2}}$ were measured even though the maximum acceleration of the Turtlebot3 is around 0.2$\frac{m}{s^{2}}$; however, the algorithm was still able to keep the error on the orientation lower than $\frac{\pi }{2}$, which is the minimum needed to at least track the orientation of the robot. The Odom–UWB modality (red) performed slightly worse than the Odom–Gyro–UWB modality (blue). The reason for this is that, during the fast short corners, the wheels of the robot slipped heavily, thus giving a completely wrong estimation of the speed and angular velocity. The gyroscope, however, was unaffected by this and, thus, could keep the uncertainty of the orientation lower, which resulted in better performance on the position as well.

The vanilla PF (purple), which uses odometry and the gyroscope, similar to the Odom–Gyro–UWB modality, shows comparable results for the orientation; however, it is less accurate on the position.

The Odom–Gyro–UWB modality improved the RMSE of the position by 28% in the LOS with four anchors, reducing it from 18 cm to 13 cm, while improving the RMSE of the orientation by 40% compared to the reference of the Gyro–Mag modality (black), reducing the RMSE from 0.2 (rad) to 0.12 (rad). As it performed the best, the influence of the number of anchors and NLOS was only shown by the Odom–Gyro–UWB modality, though similar effects could be seen with the other modalities. A smooth Variable Structure Filter (SVSF) is known for its adaptability and robustness in dynamic environments [25] and could be an alternative approach to the proposed solution. However, this requires being combined with a PF to cover the initial unknown orientation of the robot. The design and implementation of a PF of an SVSF for this particular problem could be future work.

#### 4.1.2. LOS Condition

The algorithm, for the Odom–Gyro–UWB modality (blue), seems to be unaffected by the NLOS conditions and performs similarly to the LOS condition. In contrast, the vanilla PF (purple) suffers heavily from the NLOS conditions, as can be seen in Figure 6.

Although difficult to compare to the others due to the different hardware, software, and experiments, Ref. [14] achieved an RMSE of $9.74^{\circ }$ or 0.17 (rad) on their best trajectory with five anchors in LOS conditions. Achieving the 0.12 (rad) RMSE in this paper for the Odom–Gyro–UWB modality with four anchors in the LOS shows a slight improvement in the estimation of the orientation. A major advantage of the approach in this paper is that no training is required, and the performance is proven to work in changing environments (LOS vs. NLOS experiment). A disadvantage, of course, is that the particle filter requires quite a few parameters to function. As far as the authors of this paper know, this is the only reference that did not use the magnetometer, but UWB to correct for the drift of the gyroscope in a single-tag multi-anchor setup. It is worth noting that the RMSE of the Gyro–Mag modality in [14] is $27.61^{\circ }$ or 0.48 (rad), which shows a stronger distortion for their indoor environment than can be observed in this paper and which is comparable to the RMSE of the worst behaving modality in this paper.

#### 4.1.3. Number of Anchors

The number of anchors, as expected, improved the orientation and position estimation, as is evident from Figure 7. However, the improvement is not as big as with the four-anchor case. The position improved by 18% with seven anchors, reducing the RMSE from 11 cm to 9 cm. This improvement is much less noticeable than the 28% improvement with four anchors. The orientation improved only slightly compared to the four-anchor case.

### 4.2. Online Pose Control

The algorithm is able to keep the robot on or close to the desired trajectory most of the time, as shown in Figure 8. Of course, the $180^{\circ }$ turn close to the point (0, 0) is very difficult for ego-motion sensors, and this is to be expected, especially in the case where only the Odom–UWB modality was used. It is, however, unclear why the algorithm seems to systematically lose track of the robot in the top-left corner, (−2, 2), while keeping good track of the robot in the three other corners. Possibly, UWB had some coverage issues in this corner. One can also note that, even though the algorithm loses track of the robot, it is capable of correcting for this and performs relatively stably and robustly under disturbances.

## 5. Conclusions

This paper adopted a particle filter algorithm that reduces the number of operations to fuse ego-motion modalities with UWB in such a way that the algorithm can run in real time. The algorithm estimates both the position and orientation of a robot using UWB to correct for the ego-motion drift, thereby reducing the need to use a magnetometer in indoor environments. Although a moving horizon is used as one of the states of the particle filter, the true orientation and position are calculated as well and used. The particle filter was tested offline in different conditions and with different ego-motion modalities. The best results were obtained when combining the odometry with the gyroscope for the ego-motion. In this case, an RMSE of 13 cm on the position and 0.12 (rad) on the orientation was achieved with four anchors in LOS conditions.

The algorithm was also tested and showcased using direct feedback to the trajectory control, where it performed stably on the largest part of the trajectory. Furthermore, even when it loses track, the algorithm is capable of getting the robot back on track, hence proving its robustness.

## Figures and Tables

**Table 1:** Comparison of algorithms and methods used for real-time pose control of a robot combining ego-motion modalities with UWB. Our method uses a single tag, does not use a magnetometer, which is unreliable indoors, and does not require additional calibration after the initial UWB anchor install and calibration phase.

Reference (Year)	Method	Single Tag	Orientation without Magnetometer	No Additional Calibration
[7] (2020)	Extended Kalman filter	✓		✓
[8] (2020)	Long Short-Term Memory neural network	✓		✓
[9] (2021)	Gradient Descent Optimizer	✓		✓
[10] (2021)	Gradient Descent Optimizer	✓		✓
[11] (2019)	Particle filter	✓		✓
[12] (2018)	Multi-tag reference frame		✓	✓
[13] (2021)	Extended Kalman filter	✓		✓
[14] (2021)	Antenna shape with pre-trained Gaussian process model	✓	✓	
[15] (2018)	Multi-tag reference frame		✓	✓
ours	Moving horizon particle filter	✓	✓	✓

**Table 2:** Table of variables.

Notation	Description
$x_{m}^{r}$, ${\dot{x}}_{m}^{r}$, ${\ddot{x}}_{m}^{r}$	Position, velocity, and acceleration of the robot expressed in reference frame *r* for modality *m*.
$w_{m}^{r}$	Angular velocity of the robot.
$R^{r_{1},r_{2}}$	Rotation matrix from reference frame $r_{1}$ to $r_{2}$.
$\theta^{r_{1},r_{2}}$	Angle around *z*-axis from the reference frame $r_{1}$ to $r_{2}$ in the planar case.
$B_{m}^{r}$	Bias vector in case the gyroscope and/or accelerometer are used.
$e_{m}^{r}$	Noise vector expressed in reference frame *r* for modality *m*. Assumed to be zero mean Gaussian.
$\sigma_{v,m}^{2}$	Variance of a zero mean Gaussian of a measured or derived variable *v* of modality *m*.
$A_{v}$	Transformation matrix for variable *v*.
$O^{r}$	Origin of a reference frame.
t, $t_{res}$, $\Delta t_{MH}$	The *current time*, the *reset time*, and the *moving horizon duration*, respectively.
	Moving average of the variable *v* over a period
$\bar{v}$	equal to the moving horizon duration $\Delta t_{MH}$ starting from the reset time $t_{res}$
	$=\frac{1}{\Delta t_{MH}}\int_{t_{res}}^{t_{res}+\Delta t_{MH}}v\;dt$.

**Table 3:** Table of UWB anchor locations in the navigation frame. The last three anchors, denoted by A${\#}^{*}$, are used in the seven-anchor setup.

Anchor ID	$x^{n}$ [m]	$y^{n}$ [m]	$z^{n}$ [m]
A1	3.86	−5.31	0.44
A2	3.98	5.42	2.86
A3	−3.99	−5.29	2.65
A4	3.86	−5.31	2.66
A5*	−4.05	5.45	0.41
A6*	−3.99	−5.29	0.44
A7*	−4.05	5.45	2.62

**Table 4:** Table of sensor models.

Sensor Model	
Accelerometer	InvenSense MPU-9250 from TDK InvenSense, San Jose, CA, USA
Gyroscope	InvenSense MPU9250 from TDK InvenSense, San Jose, CA, USA
Magnetometer	InvenSense MPU9250 from TDK InvenSense, San Jose, CA, USA
Wheel encoders	DYNAMIXEL XL430-W250, from ROBOTIS Co., Ltd., Seoul, Republic of Korea
UWB	Wi-Pos [24] (DW1000 from Qorvo, Greensboro, NC, USA)

**Table 5:** Table of modalities (mods.).

**Ego-Motion Modality Used in the Particle Filter**	
Odom–UWB (in red)	Only odometry is used for the ego-motion prediction. Correction is performed with UWB.
Odom–Gyro–UWB (in blue)	Odometry is used for the translation in the body frame, while the gyroscope provides the rotational motion during the prediction. Correction is performed with UWB.
IMU–UWB (in green)	Accelerometers are used for the translation in the body frame, while the gyroscope provides the rotational motion during the prediction. Correction is performed with UWB. (For the IMU, 1000 particles were used instead of 100.)
**Reference Modality to Compare with**	
UWB (in black)	The UWB pose is directly calculated from the ranges. It does not provide orientation.
Gyro–Mag (in black)	Gyroscope corrected with a magnetometer for orientation. Widely used in most applications today.
Vanilla PF (in purple)	A vanilla particle filter implementation using the odometry and gyroscope as sources similar to the Odom–Gyro–UWB modality. However, this particle filter does not use the moving horizon, as is proposed in this paper.

**Table 6:** Tables of algorithm parameters.

**Time Parameters (SF = Sampling Frequencies)**	
$\Delta t_{MH}$	1 s
UWB SF	5 Hz
IMU SF	50 Hz
Odom SF	25 Hz
**UWB Uncertainty Parameters (#A = # Anchors) **	
$\sigma_{x_{uwb}^{n}}$ 7 Anchors LOS	0.25 m
$\sigma_{x_{uwb}^{n}}$ 4 Anchors LOS	0.40 m
$\sigma_{x_{uwb}^{n}}$ 4 Anchors NLOS	0.45 m
**Ego-Motion Uncertainty Parameters**	
$\sigma_{{\dot{x}}_{odom}^{b}}$	0.2 $\frac{m}{s}$
$\sigma_{{\ddot{x}}_{acc}^{b}}$	1. $\frac{m^{2}}{s}$
$\sigma_{w_{odom}^{b}}$	0.5 $\frac{rad}{s}$
$\sigma_{w_{gyro}^{b}}$	0.2 $\frac{rad}{s}$

**Table 7:** Table of the error on the orientation in rad. The proposed method, Odom–Gyro–UWB modality, compares well with the vanilla PF and the Gyro–Mag modality, as can be seen in the top portion of the table. However, it seems to suffer more from errors beyond the P90, which is evident by the almost three-times higher maximum error. In the NLOS, the proposed solution is almost not affected by the NLOS, while the vanilla PF suffers heavily from this, as shown in the middle portion of the table. The orientation accuracy increases with the number of anchors as expected, as is evident from the bottom portion of the table.

**Error on the Orientation in Rad**						
**Modalities Analysis**						
Modality	# of Anchors	NLOS	P50	P90	RMSE	Max Error
Odom–Gyro–UWB	4		0.06	0.16	0.12	1.0
Odom–UWB	4		0.11	0.38	0.42	2.9
IMU–UWB	4		0.43	0.75	0.49	0.95
Vanilla PF	4		0.06	0.17	0.09	0.37
Gyro–Mag	NA	NA	0.18	0.31	0.20	0.36
Only gyroscope	NA	NA	1.5	2.7	1.7	3.14
Only odometry	NA	NA	1.3	2.5	1.5	3.14
**Error on the Orientation in Rad**						
**LOS vs. NLOS**						
Modality	# of Anchors	NLOS	P50	P90	RMSE	Max Error
Odom–Gyro–UWB	4		0.06	0.16	0.12	1.0
Odom–Gyro–UWB	4	✓	0.06	0.14	0.11	0.88
Vanilla PF	4		0.17	0.09	0.37	
Vanilla PF	4	✓	0.13	0.29	0.14	3.05
Gyro–Mag	NA	NA	0.18	0.31	0.20	0.36
**Error on the Orientation in Rad**						
**Number of Anchors**						
Modality	# of Anchors	NLOS	P50	P90	RMSE	Max Error
Odom–Gyro–UWB	4		0.06	0.16	0.12	1.0
Odom–Gyro–UWB	7		0.05	0.12	0.08	0.32
Gyro–Mag	NA	NA	0.18	0.31	0.20	0.36

**Table 8:** Table of the error on the position in m. The proposed method, Odom–Gyro–UWB, outperforms all other methods, as can be seen in the top portion of the table. In the NLOS, the proposed solution suffers slightly with one mm bigger RMSE, while the vanilla PF suffers heavily from this with five mm bigger RMSE, as shown in the second portion of the table. The increased number of anchors slightly improves the position accuracy; however, the effect is less strong with the increased number of anchors, as shown in the bottom portion of the table.

**Error on the Position in m**						
**Modalities’ Analysis**						
Modality	# of Anchors	NLOS	P50	P90	RMSE	Max Error
Odom–Gyro–UWB	4		0.11	0.19	0.13	0.36
Odom–UWB	4		0.14	0.31	0.21	1.1
IMU–UWB	4		0.20	0.41	0.27	1.0
Vanilla PF	4		0.13	0.41	0.18	0.72
UWB	4		0.13	0.27	0.18	0.71
Gyro–Mag–IMU	NA	NA	22 × 10^3^	45 × 10^3^	27 × 10^3^	NA
Gyro–Mag–Odometry	NA	NA	5.3	8.2	5.6	NA
**Error on the Position in m**						
**LOS vs. NLOS**						
Modality	# of Anchors	NLOS	P50	P90	RMSE	Max Error
Odom–Gyro–UWB	4		0.11	0.19	0.13	0.36
Odom–Gyro–UWB	4	✓	0.14	0.21	0.14	0.37
Vanilla PF	4		0.13	0.41	0.18	0.72
Vanilla PF	4	✓	0.18	0.52	0.23	0.79
UWB	4		0.13	0.27	0.18	0.71
UWB	4	✓	0.13	0.29	0.18	0.65
**Error on the Position in m**						
**Number of Anchors**						
Modality	# of Anchors	NLOS	P50	P90	RMSE	Max Error
Odom–Gyro–UWB	4		0.11	0.19	0.13	0.36
Odom–Gyro–UWB	7		0.07	0.15	0.09	0.28
UWB	4		0.13	0.27	0.18	0.71
UWB	7		0.09	0.17	0.11	0.39

## Data Availability

Dataset available on request from the authors.
